# Dr. Warren’s Address before the American Medical Association of Cincinnati

**Published:** 1851-04

**Authors:** 


DR. WARREN'S ADDRESS BEFORE THE AMERICAN MEDICAL ASSOCIATION AT CINCINNATI.

We have just received, from the hands of its venerable author, a copy of this interesting address. There are many portions of it that have given us a great deal of satisfaction, but we regret, that in the history of modern surgery, Dr. Warren, when addressing such a body as the American Medical Association, should have confined his history almost entirely to Europe and to the vicinity of Boston. We wish no undue importance to be assigned anywhere, or under any circumstances to Western surgery, but we cannot think it right to slur over its claims without even an allusion to its merits. The name that has given imperishable honor to American lithotomy, which has given the most exalted character to various departments of surgery is not even hinted at in the address of an American surgeon, who speaks largely of lithotomy and calculous diseases!

With the exception of this defect, we admire the general tenor of Dr. Warren's address. It is beautifully printed, and as neatly published as any European house could have done it. B.

